# Copper-catalyzed dicarbonyl stress in NAFLD mice: protective effects of Oleuropein treatment on liver damage

**DOI:** 10.1186/s12986-022-00641-z

**Published:** 2022-02-11

**Authors:** Silvano Junior Santini, Giovanni Tarantino, Antonella Iezzi, Anna Alisi, Clara Balsano

**Affiliations:** 1 Department of Life, Health and Environmental Sciences MESVA, University of L’Aquila, Piazza S. Salvatore Tommasi 1, 67100 Coppito, L’Aquila Italy; 2 Francesco Balsano Foundation, Via Giovanni Battista Martini 6, 00198 Rome, Italy; 3 Department of Clinical Medicine and Surgery, Federico II University Medical School of Naples, Naples, Italy; 4 Research Unit of Molecular Genetics of Complex Phenotypes, Bambino Gesù Children’s Hospital, IRCCS, Viale San Paolo 15, 00146 Rome, Italy

**Keywords:** NAFLD, MAFLD, Oleuropein, Dicarbonilyl stress, Copper, Sex, Nutriaceutical compounds

## Abstract

**Background:**

Nonalcoholic fatty liver disease (NAFLD) or more appropriately, metabolic associated fatty liver disease (MAFLD), is the hepatic manifestation of metabolic syndrome. An imbalance of copper homeostasis has been described in the progression of NAFLD/MAFLD toward NASH/MASH. We were interested in understanding whether the chelating activity of Oleuropein (Ole) was able to improve the copper accumulation and the related pro-oxidant and glycative damage in the liver of mice fed HFD.

**Methods:**

Twelve C57BL/6J mice fed normal diet (ND) or high-fat diet (HFD) for 16 weeks and then thirty two female and male mice fed ND or HFD for 8 weeks adding Ole for the following 8 weeks were studied.

**Results:**

Altered expression of copper-trafficking genes and proteins (CTR1, CTR2, ATP7B, COX17, CCS, and ATOX1) induced imbalance of copper homeostasis combined with an increase in dicarbonyl stress in the liver of HFD fed mice. Interestingly enough, glyoxalase system was improved by Ole administration and the Ole related protective effects differ in the two sexes of mice.

**Conclusions:**

Our study highlights the role of the dicarbonyl stress in the pathogenesis of NAFLD and suggests Ole as a natural copper chelator to prevent the liver damage induced by methyglyoxal pathway derangement.

**Supplementary Information:**

The online version contains supplementary material available at 10.1186/s12986-022-00641-z.

## Background

NAFLD (Nonalcoholic fatty liver disease), one of the major forms of chronic liver disease, is considered the hepatic manifestation of the metabolic syndrome (MetS), thus is currently called metabolic associated fatty liver disease (MAFLD) [1]. The global prevalence of NAFLD/MAFLD is estimated to be 24%; the highest rates were reported from South America and the Middle East, followed by Asia, the United States and Europe [2]. Nonalcoholic steatohepatitis (NASH), or MASH (metabolic-associated steatohepatitis) using the new terminology, is a form of NAFLD/MAFLD in which a buildup of fat in the liver causes liver inflammation and damage and may lead to cirrhosis and/or hepatocellular carcinoma (HCC) [3].

The alteration of hepatic glycolysis needs growing attention because of its interaction with the progression of NAFLD/MAFLD liver disease [4]. The hepatic metabolic homeostasis (glycolysis, gluconeogenesis, lipogenesis, and fatty acid oxidation) can be disrupted by various adverse conditions, one of them is the intratissue excess of copper, deeply implicated in the formation of reactive carbonyl compounds [5–7]. Accordingly, it has been highlighted enhanced glycolytic activity and higher lactate levels in patients with NAFLD/MAFLD or NASH/MASH [8].

High glycolytic fluxes commonly are associated with the overproduction of a dicarbonyl compound known as methylglyoxal (MG) [9]. Methylglyoxal is an α-oxoaldehyde present mostly in the mono and dihydrate forms in aqueous solution [10]. Methylglyoxal can derive from enzymatic or non-enzymatic reactions of different metabolic pathways. It is produced in l-threonine metabolism, catabolism of the ketone bodies acetoacetate and acetone and as a by-product of glycolysis [11–13].

Furthermore, MG is a cytotoxic and pro-apoptotic molecule whose deleterious action within cells is known to proceed through dicarbonyl stress and reactive oxygen species (ROS) [14].

Two enzymes belong to the glyoxalase system: glyoxalase I and II (GLO I and II). It is important to underline that the glyoxalase system, to be efficient, requires catalytic amounts of reduced glutathione (GSH) [15]. GLO I is present in the cytosol and represents the first line of defense, but the rate-limiting of the glyoxalase system is related to GLO II [9].

An altered homeostasis of copper (Cu) has been observed in all the different stages of NAFLD/MAFLD. In particular, recent literature indicates that serum copper concentration is increased in NAFLD/MAFLD patients transitioning from cirrhosis to HCC [16]. Copper is an essential micronutrient for living organisms and acts as a cofactor in many essential enzymes (i.e.: cytochrome C oxidase in mitochondria and Cu–Zn-dependent superoxide dismutase in cytosol) [17]. The main protein responsible for the influx of reduced copper ions across cell membranes is CTR1 (Copper transport 1) [18]. After that, copper is chelated by metallothioneins in the cytoplasm and carried to specific proteins by bounding to Cu chaperones for being released or used for glutathione reduction [19]. Three chaperones for copper have been identified: CCS (Copper chaperone for superoxide dismutase), involved in the folding of copper/zinc superoxide dismutase 1 (SOD1); COX17 (copper chaperone for cytochrome c oxidase), a highly conserved protein which influences the recruitment and incorporation of copper ions into mitochondrial cytochrome C oxidase; ATOX1 (Human antioxidant protein 1), which plays a key role in copper homeostasis in supplying copper from the cytosol to transporters ATPase Copper Transporting Alpha and Beta (ATP7A and ATP7B) [20–23]. The last two Cu-transporters ATP7A and ATP7B allow the passage of copper among the cytosol and organelles and regulate the efflux of excess of copper out of the cell [24]. These two proteins are tissue-specific: ATP7A is present in all tissues except in the liver, where ATP7B is strongly expressed [25, 26].

The principal health constituent of Mediterranean diet is olive oil. Olive oil is rich in Ole, the main polyphenol compound present in the green olives and olive leaves [27]. Our previous works have shown that Ole is able to decrease the accumulation of fat and to counteract the damage related to oxidative stress. In particular, in the same mouse model, Ole was able to activate the autophagic process through AMPK-dependent phosphorylation of ULK1 at Ser555 and to improve the activity of SOD2 and SOD1. For the latter the antioxidant effect was achieved by its delocalization in the nucleus [28, 29]. Interestingly enough, it has been demonstrated that Ole is able to complex and chelate copper, in vitro [30]. Furthermore, oxidative damage has been associated to chronic exposure to excess copper caused by metabolic disorders and liver is an important copper storage organ in mammals [28, 31].

Our study seeks to evaluate if Ole, a natural copper chelator, may improve the imbalance of copper homeostasis and the associated glycative liver damage in mice fed high fat diet (HFD).

## Methods

### Mice experimental protocol

Twelve C57BL/6J mice, 2 months old, were purchased from Charles River Laboratories (Calco, Lecco, Italy) and housed individually in a temperature-controlled (21 ± 1 °C) room, on a 12 h light–dark cycle. The animals had free access to food and water. Mice were randomly divided into two different groups (6 animals per group), fed for 16 weeks with one of the following types of diet: (i) HFD that was characterized by the presence of protein kcal% 15.2, carbohydrate kcal% 42.7 (34.5% sucrose) and fat kcal% 42.0 (TD.88137, Harlan Laboratories Indianapolis, IN, USA; (ii) normal diet (ND) that was characterized by protein kcal% 24.0, carbohydrate kcal% 58.0 and fat kcal% 18.0 (Teklad Global 2018, Harlan Laboratories Indianapolis, IN, USA; ND group) [32].

For the second part of the study, we used 32 male and female ND and HFD fed C57BL/6J mice, treated or not with 0.03% of Ole, the most commonly used concentration [33, 34]. Food and water intake was recorded daily, each mouse consumed about 0.5 mL of water per day. The daily dose consumed corresponds approximately to an intake of 5.6 mg/kg body weight for oleuropein, which corresponds to the daily dose used in humans (0.7–3.5 mg/kg) [35]. Mice were divided into 4 groups (4 male and 4 females for each group): ND, ND + Ole, HFD and HFD + Ole. We used the experimental protocol to feed and treat mice as reported above. Ole was dissolved in water according to the manufacturer's instructions and administered, after the first 8 weeks, at the final concentration of 0.03% by oral gavage. After 16 weeks, blood was collected from the abdominal veins after fasting for 12 h and liver was removed, washed in ice-cold PBS, weighted and photographed. Then, samples were formalin-fixed for immune histological analysis or immediately frozen and stored at − 80 °C until use for subsequent analysis.

Serum levels of triglycerides, alanine aminotransferase (ALT), aspartate aminotransferase (AST), total cholesterol, were measured using an Architect platform (Abbott Laboratories, Chicago, IL) and commercially available kits, according to the manufacturer’s instructions. Animals’ body weights and biochemical parameters were reported in Additional file 1: Table S1.

All animal protocols were in accordance with the Guide for the Care and Use of Laboratory Animals and approved by the Institutional Animal Care and Use Committee at the University of Florence, Italy (178/2013B).

### Cell culture

The human hepatoma cell line, HepG2, (ATCC cat. HB-8065) were purchased from American Type Culture Collection (ATCC, Manassas, VA). HepG2 were cultivated as previously described by Santini and colleagues [29]. Long-chain FAs, palmitic acid (PA; 16:0) and oleic acid (OA; 18:1) (Sigma-Aldrich, Milan, Italy) were dissolved in methanol (MetOH) 99%. Steatosis was induced as previously described by Ricchi et al. [36]. Briefly, culture medium was supplemented with a solution of FAs (0.16 mM PA and 0.33 mM OA). Cells incubated with MetOH were considered as control.

After 24 h, Oleuropein was added to HepG2 cells at the following concentrations: 50, 100 and 200 μM. Oleuropein (Sigma-Aldrich cat. 12247, Milan, Italy).

### Histological analysis

Specimens were formalin-fixed, paraffin-embedded and sectioned in order to assess the histological features by hematoxylin and eosin (H&E) staining analysis, as described by Cardiff and co-workers [37].

### Determination of copper levels and biochemical parameters

HepG2 cells and liver tissue were lysate in PBS by sonication. Lysates samples and mice serum were diluted 1:2 (v:v) with 65% nitric acid (ApplicChemPanreac). Copper content was assayed by atomic absorption spectroscopy using an Analyst 300 instrument equipped with a graphite furnace with platform (HGA800) and an AS-72 autosampler (Perkin-Elmer, Waltham, MA, USA). And the results of serum copper content obtained were given as µg/dL serum and protein concentration was used for normalization of intracellular and tissue copper content. Serum Total cholesterol, triglyceride (TRIG), high-density lipoprotein cholesterol (HDLc), low-density lipoprotein cholesterol (LDL-c) and ALT and AST levels were analyzed by using an automatic chemical analyzer (BS-120 Chemistry Analyzer; Mindray). All assays were conducted in quadruplicate using fresh serum.

### RNA extraction, cDNA synthesis and real-time quantitative PCR (qPCR)

Total RNA extraction was performed using QIAzol Lysis Reagent (Qiagen Inc.) according to the manufacturer’s instructions and 1 µg was reverse transcribed using First Strand cDNA Synthesis Kit (OriGene) according to the manufacturer’s protocol. The cDNA was used (1:100) for the quantitative PCR reactions using the Applied Biosystems 7500 system (ThermoFisher Scientific, Inc., Rockford, IL) and the SensiFast SYBR-based kit (cat. Bio 92005, Bioline, London, UK) and Custom primers were synthetized by IDT Integrated DNA Technologies, Inc. (Coralville, IA, USA). The following primers were used: β-actina [38] Forward primer (Fw) 5′-GAGACCTTCAACACCCCAGC-3′ Reverse (Rv) 5′-ATGTCACGCACGATTTCCC-3′; ctr1 [39] Fw 5′-CATGATGATGATGCCTATGACC-3′ Rv 5′-CAGCATCTGCTGCCCAAC-3′; ctr2 [40] Fw 5′-AACTTCAGACAATAGGACCCGCCT-3′ Rv 5′-TAGGACATGACAGCCAGCATCACA-3′; atp7B [41] Fw 5′-CAGCCAGAGCCATTGCTACTCA-3′ Rv 5′-GAAGGCAGTACCTCCGCAAAGA-3′; Cox17 [40] Fw 5′-CTCAGGGTAGTCGGAGTTTG-3′ Rv 5′-AAGTTCTCCAAAGAACTTCC-3′; ccs [41] Fw 5′-CGGCCTAGGCAGTGACAACA-3′ Rv 5′-AGTCGTCTGCACCAACACCATC-3′; atox1 [41] Fw 5′-TCAACAAGCTGGGAGGAGTG-3′ Rv 5′-ACATGGAAGCTTGCAGGGAG-3′; glo I [38] Fw 5′-GATTTGGTCACATTGGGATTGC-3′ Rv 5′-TTCTTTCATTTTCCCGTCATCAG-3′; glo II [38] Fw 5′-GGGAACGAGAAGCTGGTGAA-3′ Rv 5′-CCGAAGTATGGCAGGGTGTT-3′; nrf2 [42] Fw 5′-CTGAACTCCTGGACGGGACTA-3′ Rv 5′-CGGTGGGTCTCCGTAAATGG-3′. Gene expression was calculated as described by Livak et al. [43]. The assays were performed in quadruplicate.

### Glutathione assay

Total glutathione (tGSH) and oxidized glutathione (GSSG) levels were determined by using a glutathione assay kit (cat 703002, Cayman Chemical, Ann Arbor, USA) based on Ellman's reagent [44], as described by the manufacturer. The concentration of tGSH and GSSG were determined using calibration curves that were obtained from reactions containing either pure GSH or pure GSSG standards. Results were given as tGSH over GSSG ratio. Measurements were carried out in quadruplicate.

### Evaluation dicarbonyl stress

The damage due to dicarbonyl stress was evaluated using the OxiSelect Methylglyoxal Competitive ELISA Kit (cat. STA-811, Cell Biolabs, INC, San Diego, USA), an enzyme immunoassay developed for rapid detection and quantification of protein-MG-H1 (methyl-glyoxal-hydro-imidazolone) adducts. The amount of MG adduct in protein samples is determined by comparing its absorbance with that of a standard MG-BSA curve of known concentration.

### Western immunoblot analysis

Liver tissues were homogenized in RIPA buffer plus protease and phosphatase inhibitors (Sigma-Aldrich), as suggested by the manufacturer. Denatured protein samples were analyzed through SDS–PAGE and probed with different primary antibodies and horseradish peroxidase–conjugate secondary antibody and detected by ECL ECL Star Enhanced Chemiluminescent Substrate plus (Euroclone). Abcam (Cambridge, UK) provided the following primary antibodies: anti-GLO II (ab154108,); anti-NRF2 (ab76026). The anti-GLO I (sc-67351); anti-CCS (sc-20141); anti CTR1 (sc-66847); anti-ATP7B (sc-373964); anti-COX17 (sc-100521) was purchased from Santa Cruz Biotechnology, Inc, Dallas, TX, USA) and anti-CTR2 (cat. PA5-22961) from Invitrogen (Carlsbad, CA, USA) and anti-ATOX1 (cat. MBS4154812) from Mybiosource (San Diego, CA, USA). The HRP-conjugated goat anti-rabbit IgG secondary antibody (PI1000) was purchased from Vector Laboratories (Burlingame, CA, USA). The peroxidase-conjugated anti-mouse secondary antibody (A9044) was purchased from Sigma-Aldrich (Milan, Italy). The images of the specific immune complexes were revealed, acquired and analyzed by using Enhanced Chemi-luminescent Substrate Kit (cat. EMP001005, Euroclone, Milan, Italy).

### Liver extract preparation for glyoxalases activity assessments

Livers were lysed (250 mg/mL) in 100 mM phosphate buffer (pH 7), containing 1.5 mM dithiothreitol (DTT) and 1 mM EDTA for glyoxalase 1 and glyoxalase 2 enzyme activities. The homogenates were centrifuged, and the supernatants used for assessments of enzymatic activities and protein concentrations were determined using the Bradford Protein Assay (cat. 500-0006 Bio-Rad, Hercules, CA, USA) using BSA as the standard All readings were carried out in quadruplicates, using a Lamba25 UV–Vis spectrophotometer (PerkinElmer, Waltham, MA, USA).

### Glyoxalase I (GLO I) and glyoxalase II (GLO II) enzymatic activity

The GLO I (EC 4.4.1.5) activity was determined by recording at 240 nm the appearance of (R)-S-lactoylglutathione in a 1 mM GSH (cat. G4251, Sigma-Aldrich) and 2 mM methylglyoxal (cat. M0252, Sigma-Aldrich) reaction mixture, as described [45]. One unit of enzyme activity was defined as 1 μmol of (R)-S-lactoylglutathione formed/min at 25 °C. Measurements were carried out in quadruplicate.

The GLO II (EC 3.1.2.6) activity was assayed by following nm the disappearance of 0.3 mM (R)-S-lactoylglutathione (cat. L7140, Sigma-Aldrich) at 240 nm, as described by Guha [46]. One unit of enzyme activity was defined as 1 μmol of lactoylglutathione hydrolyzed/min at 25 °C. Measurements were carried out in quadruplicate.

### Statistical analysis

Results were expressed as mean ± standard deviation (SD). All invitro experiments were performed in triplicate. A 2-tailed paired/un-paired Student test and the 2-tailed Mann–Whitney test and Factorial ANOVA with post-hoc Fisher LSD tests for multiple comparison were applied to analyze results of animal groups (*vs. ND mice; # vs. HFD mice; § vs. male mice). Statistical significance was assessed by *P* value (*P*) thresholds: *; #; §*P* < 0.05; **; ##; §§*P* < 0.01; ***; ###; §§§*P* < 0.001. All statistical analyses were performed with Statistical software version 10 StatSoft Software, Hamburg, Germany).

## Results

### Alteration of copper homeostasis and implication of dicarbonyl stress in liver damage of HFD mice

It is known that alterations in redox homeostasis and signaling are central in maintaining cellular homeostasis and that high levels of copper in serum and tissues correlate with high level of oxidative stress [47].

**Fig. 1:**
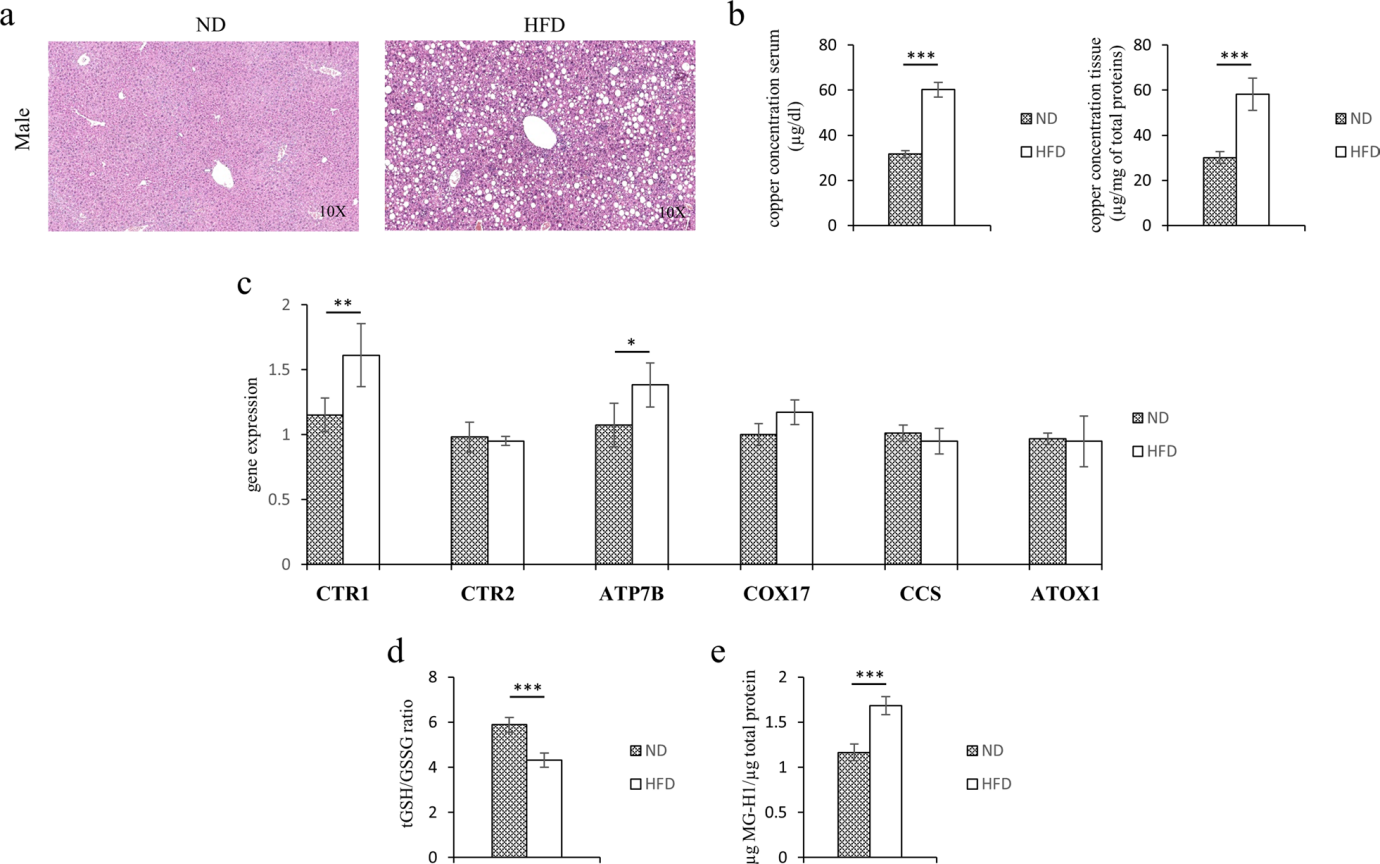
Serum and tissues copper concentrations, redox balance and MG-H1 levels in ND or HFD mice. **a** Representative photomicrographs of Hematoxylin and Eosin staining in liver sections from representative liver tissues of male mice fed with ND or HFD. Original magnification ×10. **b** Copper concentration in serum and liver samples of ND and HFD mice. **c** Transcriptional levels of CTR1, CTR2, ATP7B, COX17, CCS and ATOX1 genes. **d** Levels of reduced GSH expressed by tGSH/GSSG ratio and **d** MG-H1 (methyl-glyoxal-hydro-imidazolone) protein adducts. Values are expressed as means ± SD and data were analyzed by a t-Test analysis (*P < 0.05; **P < 0.01; ***P < 0.001)

To evaluate the role of copper in the pathogenesis of liver steatosis, we looked at the concentration of the biometal in the serum and liver tissues of a 16 weeks HFD fed mouse model. As demonstrated by Hematoxylin and eosin staining (Fig. 1a) after HFD the liver exhibited steatosis. As shown in Fig. 1b, in both the serum and liver tissues of HFD fed mice were observed higher levels of copper than in mice fed ND. In HFD fed mice, the altered concentrations of copper were associated with the altered transcriptional expression of the copper-trafficking genes: CTR1 and ATP7B (Fig. 1c).

Glutathione levels are essential for the good efficiency of antiglycative detoxification systems. In order to evaluate a possible pro-glycation effects of HFD treatment we assessed the liver amount of MG-H1 (methyl-glyoxal-hydro-imidazolone) protein adducts, one of the most sensitive dicarbonyl stress marker. Interestingly, the altered copper homeostasis was associated with the decrease of reduced GSH (expressed as total GSH/GSSG ratio) and with the increase of MG-H1 amount in the steatotic livers (Fig. 1d, e).

### In vitro and in vivo effects of Ole treatment on copper

In a previous work, we showed that Ole does not affect cell viability and is able to decrease fat accumulation in HepG2 cells [29]. Here, we checked the effect of increasing doses of Ole on copper intracellular levels of HepG2 cells in the presence of fat accumulation (FA-HepG2). Treatment with 100 and 200 mM Ole leads to a significant reduction in the intracellular copper content of FA-HepG2 (Additional file 2: Fig. S1), confirming the ability of Ole to chelate Cu.

**Fig. 2:**
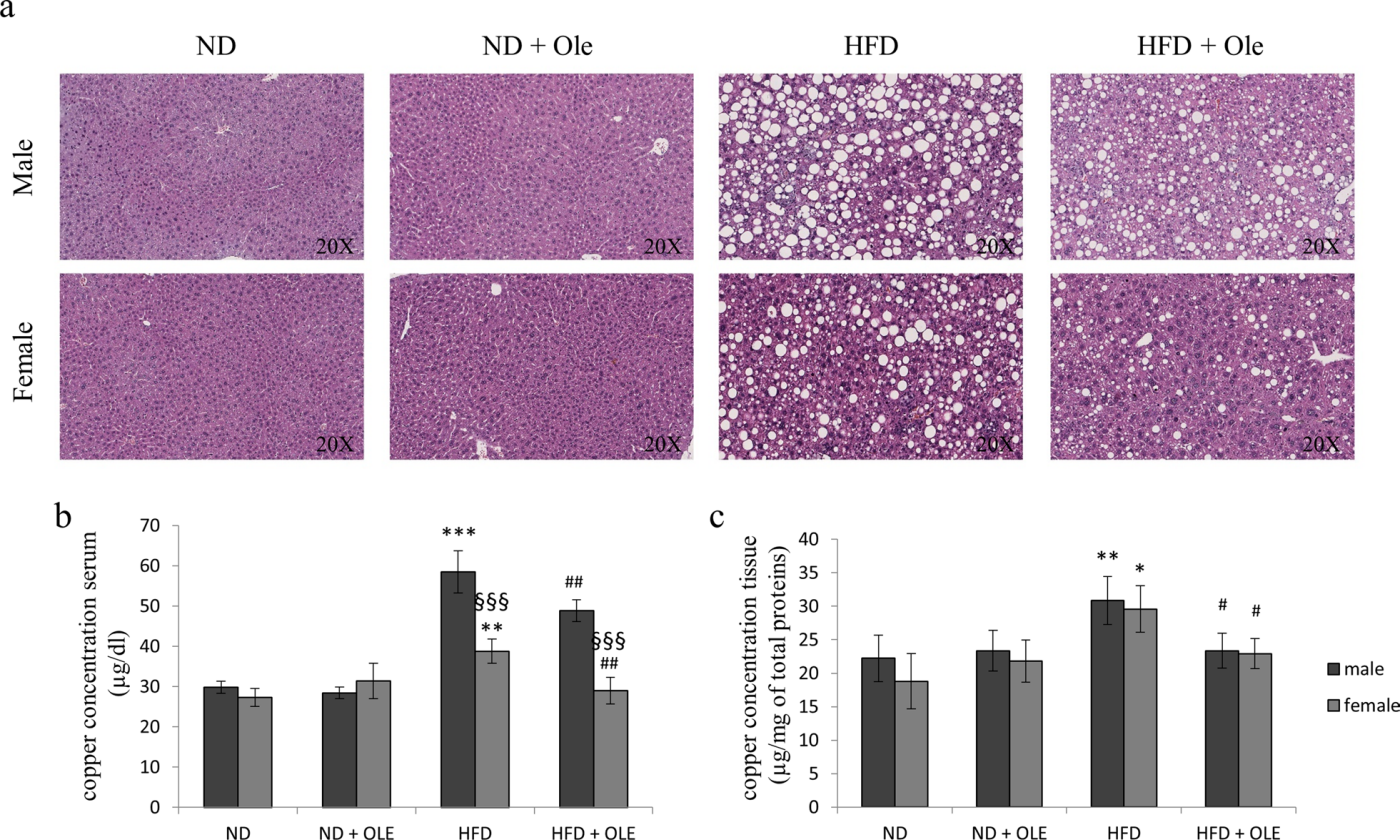
Effects of Ole on both liver histology and copper levels in ND or HFD mice. **a** Representative photomicrographs of Hematoxylin and Eosin staining in liver sections from representative liver tissues of male and female mice fed with ND or HFD in presence or absence of Ole. Original magnification ×20. Copper concentration in serum (**b**) and liver (**c**) samples of ND and HFD mice treated or not with Ole. Copper was evaluated by atomic absorption spectroscopy. The results, derived from four independent experiments, and are represented as mean ± SD. (*P < 0.05; **P < 0.01; ***P < 0.001 vs. ND mice; ^#^*P* < 0.05; ^##^*P* < 0.01 vs. HFD mice; ^§§§^*P* < 0.001 vs. male mice)

To evaluate if 0.03% Ole was able to chelate copper in vivo, we used a model of male and female mice fed ND and HFD, treated or not for 8 weeks with the natural compound [28]. Hematoxylin and eosin staining of liver tissues was performed (Fig. 2a).

After 16 weeks, male and female HFD fed mice, treated with Ole, displayed a significant less copper accumulation in the serum and liver tissue, whereas any Ole-related effects on copper concentration was highlighted in ND fed mice (Fig. 2b, c).

As expected, HFD mice, treated with Ole, showed less body and liver weight, and improved the level of transaminases and lipid profile (see Additional file 1: Table S1).

### Ole effects on proteins mediating uptake or efflux of copper

It is well known that proteins belonging to the Cu-transporter system are critical in the control of copper levels in all kingdoms of life [48]. Thus, to understand if Ole treatment was able to improve the redox imbalance by acting on copper proteins expression levels, we analyzed CTR1/CTR2, ATP7B and all Cu-chaperones proteins, which participate to the uptake and efflux of copper from cells.

**Fig. 3:**
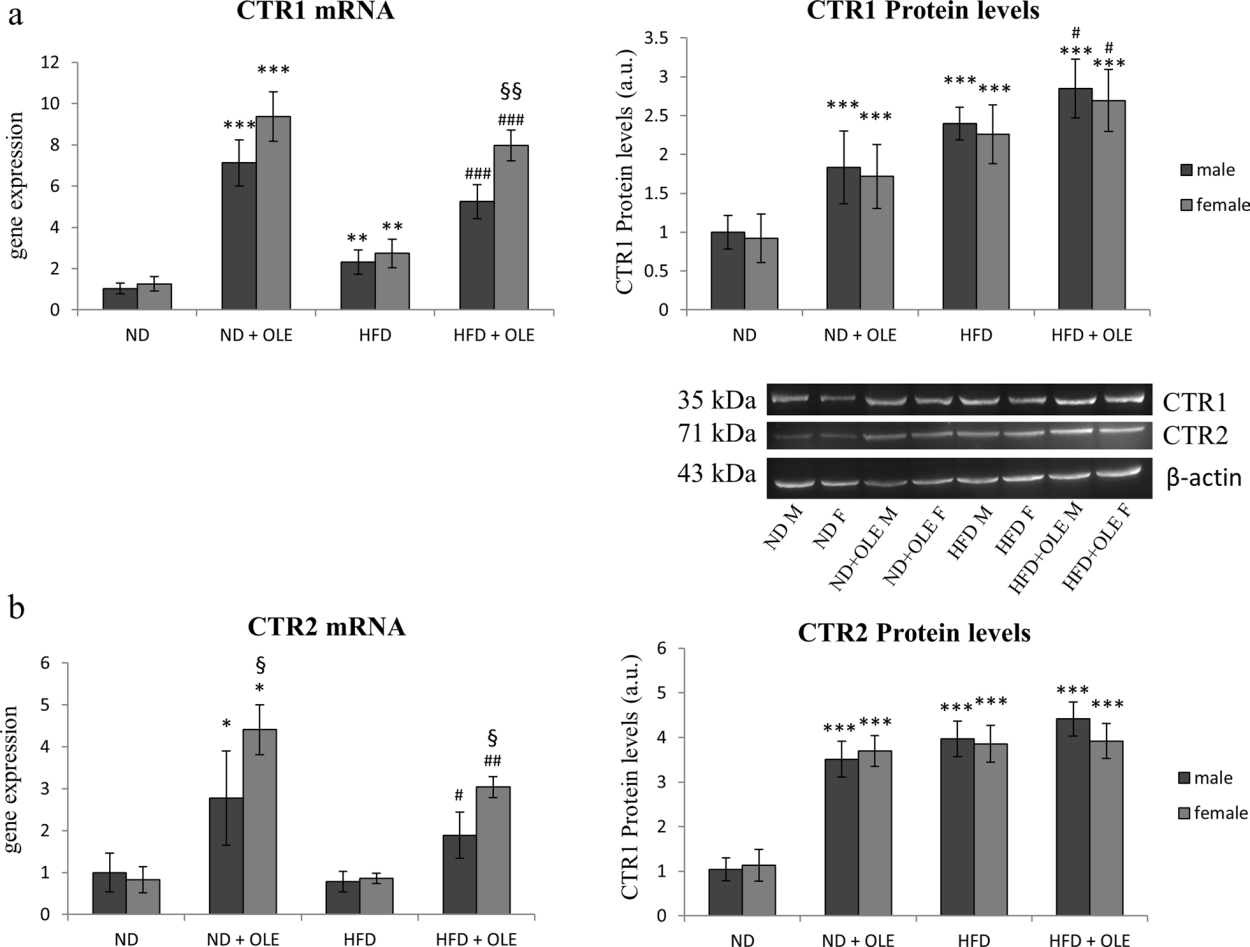
Effects of Ole Cu-transporter proteins in ND and HFD mice. RT-PCR, Western blot analysis and relative densitometry of CTR1 (**a**) and CTR2 (**b**) proteins. Values are expressed as fold mean ± SD. Data were analyzed by a factorial ANOVA with post-hoc Fisher’s tests for multiple comparison (*P < 0.05, **P < 0.01; ***P < 0.001 vs. ND mice; ^#^*P* < 0.05, ^##^*P* < 0.01, ^###^*P* < 0.001 vs. HFD mice; ^§^*P* < 0.05, ^§§^*P* < 0.01 vs. male mice)

First of all, we looked at the expression levels of CTR1 and CTR2 proteins, in ND and HFD male and female mice, treated or not with 0.03% Ole. Ole was able to upregulate the CTR1 mRNA and protein in both ND and HFD mice (Fig. 3a, b).

On the other hand, even if Ole was able to increase the protein level of CTR2 in ND mice, it failed to further increase CTR2 in HFD mice, as has been observed for the CTR1 protein (Fig. 3b). The sex of mice did not influence the Ole related effects (Fig. 3a, b).

**Fig. 4:**
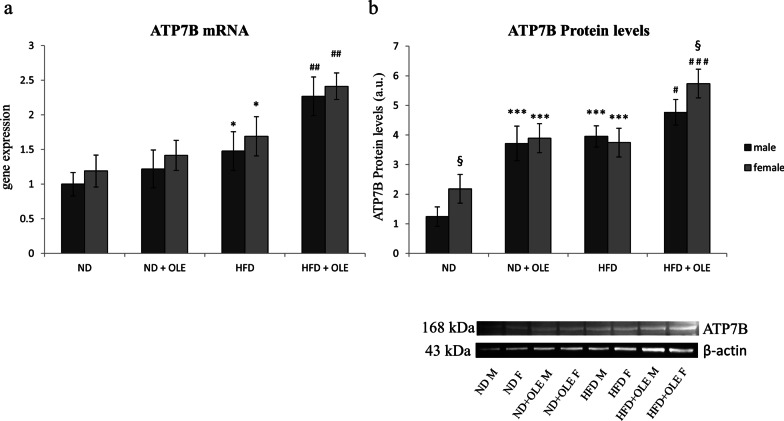
Effects of Ole treatment on ATP7B expression in ND and HFD mice mice. mRNA (**a**) and protein (**b**) levels of ATP7B are expressed as means and SD and analyzed by a factorial ANOVA with post-hoc Fisher’s tests for multiple comparison (*P < 0.05, ***P < 0.001 vs. ND mice; ^#^*P* < 0.05, ^##^*P* < 0.01, ^###^*P* < 0.001 vs. HFD mice; ^§^*P* < 0.05 vs. male mice)

In our model, ATP7B was significantly upregulated, at transcriptional levels in HFD mice, especially if treated with Ole. On the other hand, at translational levels, ATP7B displayed different expression in the two sexes of ND fed mice. Moreover, Ole treatment was able to significantly increase ATP7B either in ND or HFD fed mice (Fig. 4a, b).

### Ole effects on intracellular chaperones of copper

A greater knowledge of the role of Cu-chaperones in response to external stimuli may offer the possibility of identifying new therapeutic targets for NAFLD/MAFLD disease. Thus, we looked at the transcriptional and translational expression levels of all the Cu-chaperones in ND and HFD mice, treated or not with 0.03% Ole.

**Fig. 5:**
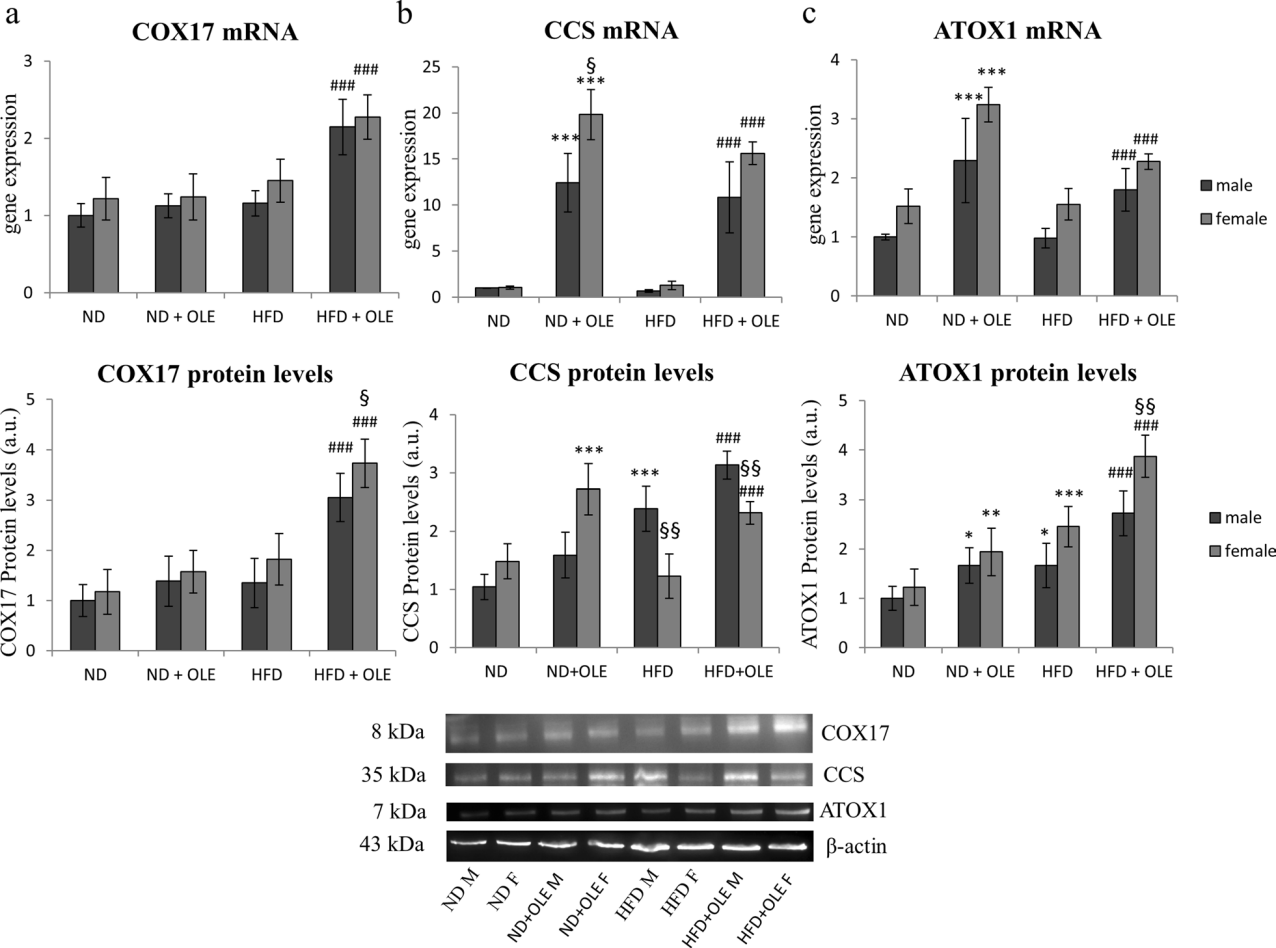
Effects of Ole on copper chaperone proteins in ND and HFD mice. RT-PCR and Western blot analysis and relative densitometry of COX17 (**a**), CCS (**b**) and ATOX1 (**c**). Values are expressed as fold mean ± SD. Data were analyzed by a factorial ANOVA with post-hoc Fisher’s tests for multiple comparison (*P < 0.05, **P < 0.05, ***P < 0.001 vs. ND mice; ^###^*P* < 0.001 vs. HFD mice; ^§^*P* < 0.05, ^§§^*P* < 0.01 vs. male mice)

In our model, COX17 was significantly increased by Ole treatment in HFD mice, let thinking that mitochondria may be a specific target of this compound in damaged tissue (Fig. 5a).

Furthermore, Ole treatment induced an increase of CCS at mRNA and protein levels, highlighting different response to the polyphenol of male respect to female mice (Fig. 5b).

Eventually, ATOX1, which donates Cu to ATP7B and combines Cu into vesicles for secretion or export of excess Cu, was significantly upregulated by Ole treatment, at mRNA and protein levels (Fig. 5c).

### Ole restores GSSG redox imbalance and dicarbonyl stress induced by HFD

It is known that alterations in redox homeostasis and signaling are central in maintaining cellular health. Moreover, high levels of copper in serum and tissues correlate with high level of oxidative stress [47].

**Fig. 6:**
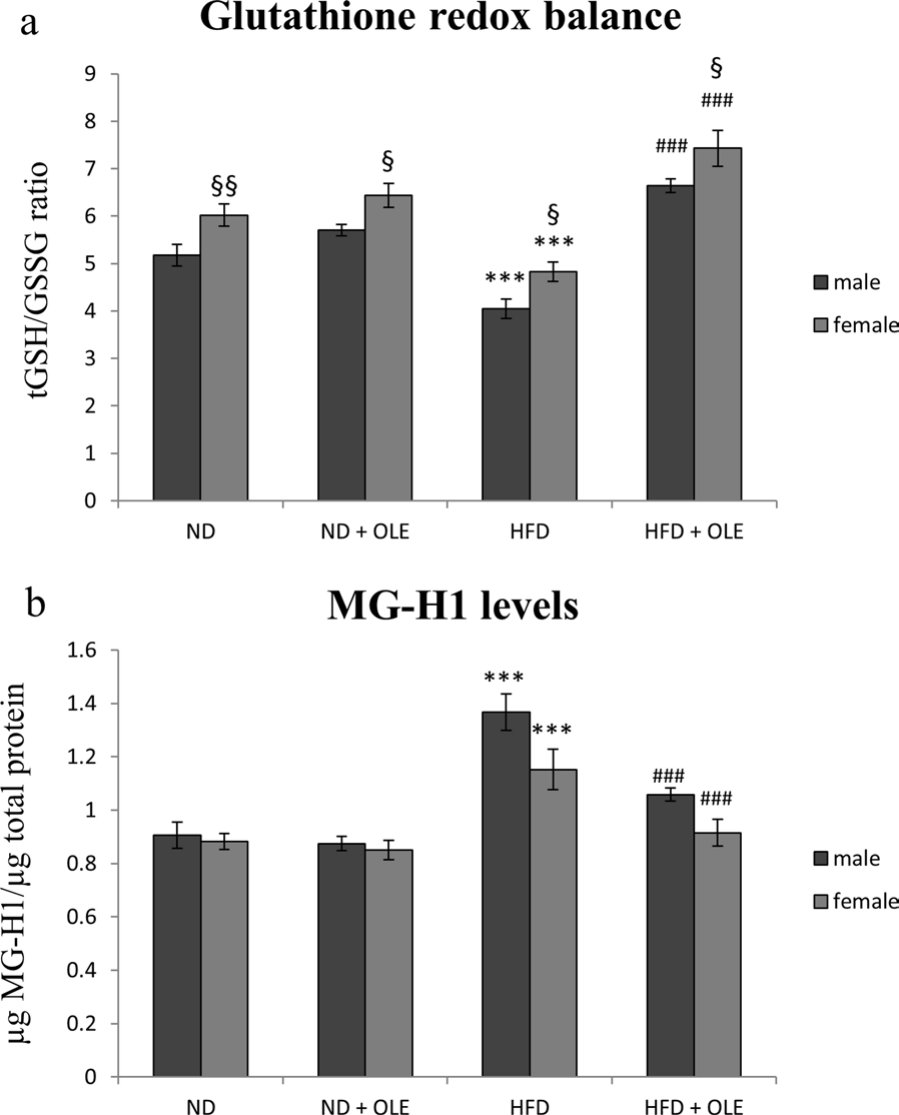
Effects of Ole treatment on glutathione redox balance and MG-H1 levels. **a** Levels of reduced GSH expressed by tGSH/GSSG ratio and **b** MG-H1 (methyl-glyoxal-hydro-imidazolone) protein adducts. Values are expressed as means ± SD and data were analyzed by a factorial ANOVA with post-hoc Fisher’s tests for multiple comparison (***P < 0.001 vs. ND mice; ^###^*P* < 0.001 vs. HFD mice; ^§^*P* < 0.05, ^§§^*P* < 0.01 vs. male mice)

Excess fat significantly reduced GSH in our HFD fed mice, therefore, we evaluated the redox imbalance after Ole treatment (Fig. 6a). Ole treatment induced an increase of GSH availability in HFD mice (Fig. 6a). At all experimental conditions GSH levels were significantly higher in female mice (Fig. 6a).

Ole administration counteracted the pro-glycation effects induced by the high fat diet intake by the decrease of MG-H1 liver amount (Fig. 6b).

### Ole improves MG detoxification

It is well known that transition metals are involved in carbonyl stress in diabetic patients [7]. Glycation-related parameters were analyzed in order to evaluate if the improvement of glycative damage by Ole was related, not only to the modulation of Cu-transporter proteins, but also to the induction of antiglycative detoxification.

**Fig. 7:**
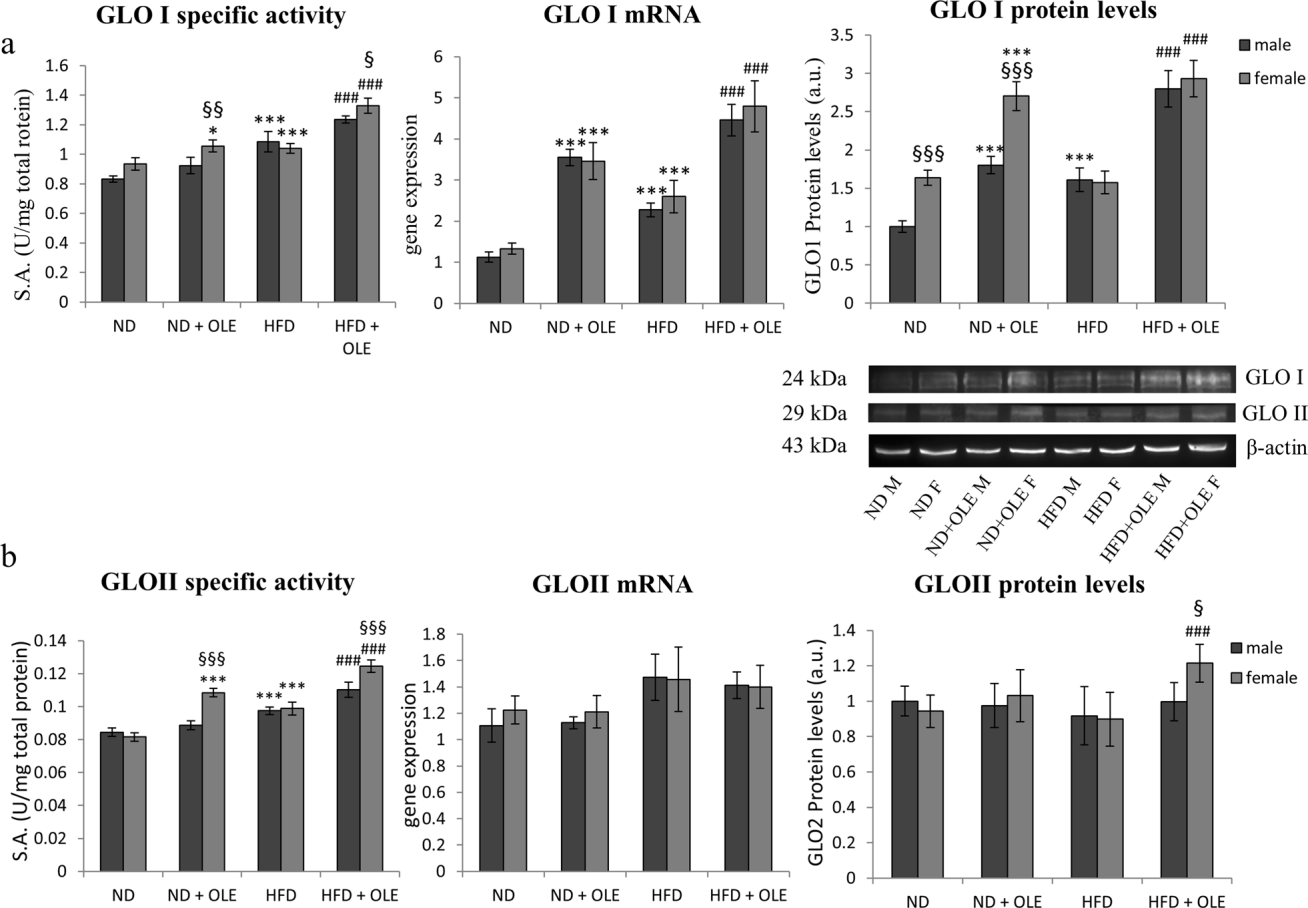
Ole and its scavenging capacity of the glyoxalase system. Enzymatic activity, transcriptional expression and protein levels of respectively glyoxalase I (GLO I) (**a**) and glyoxalase II (GLO II) (**b**). Values are expressed as fold mean ± SD. Data were analyzed by a factorial ANOVA with post-hoc Fisher’s tests for multiple comparison (*P < 0.05, ***P < 0.001 vs. ND mice; ^###^*P* < 0.001 vs. HFD mice; ^§^*P* < 0.05, ^§§^*P* < 0.01, ^§§§^*P* < 0.001 vs. male mice)

We analyzed the enzymatic activity, protein and transcript levels of the glyoxalase I and II enzymes (GLO I, GLO II), which represent the primary enzymatic defense against methylglyoxal (MG)-induced glycation. Ole treatment significantly increased the levels of transcript, protein and specific activity of GLO I, that was further helped by the Ole-related increased availability of GSH (Figs. 7a and 6a, respectively). Interestingly, in normal diet condition Ole administration led to an increase of GLO I activity, that was higher in female than to male mice (Fig. 7a).

In a specular way, we analyzed a possible involvement of Ole on the enzymatic activity, transcript and protein levels of GLO II, the rate limiting enzyme of the glyoxalase system. Remarkably, HFD mice, treated with Ole, showed a significant increase in the scavenging activity of GLO II (Fig. 7b). Again, female mice were more susceptible than males to the Ole-related beneficial effects (Fig. 7b).

### Effects of Ole on NRF2 expression

**Fig. 8:**
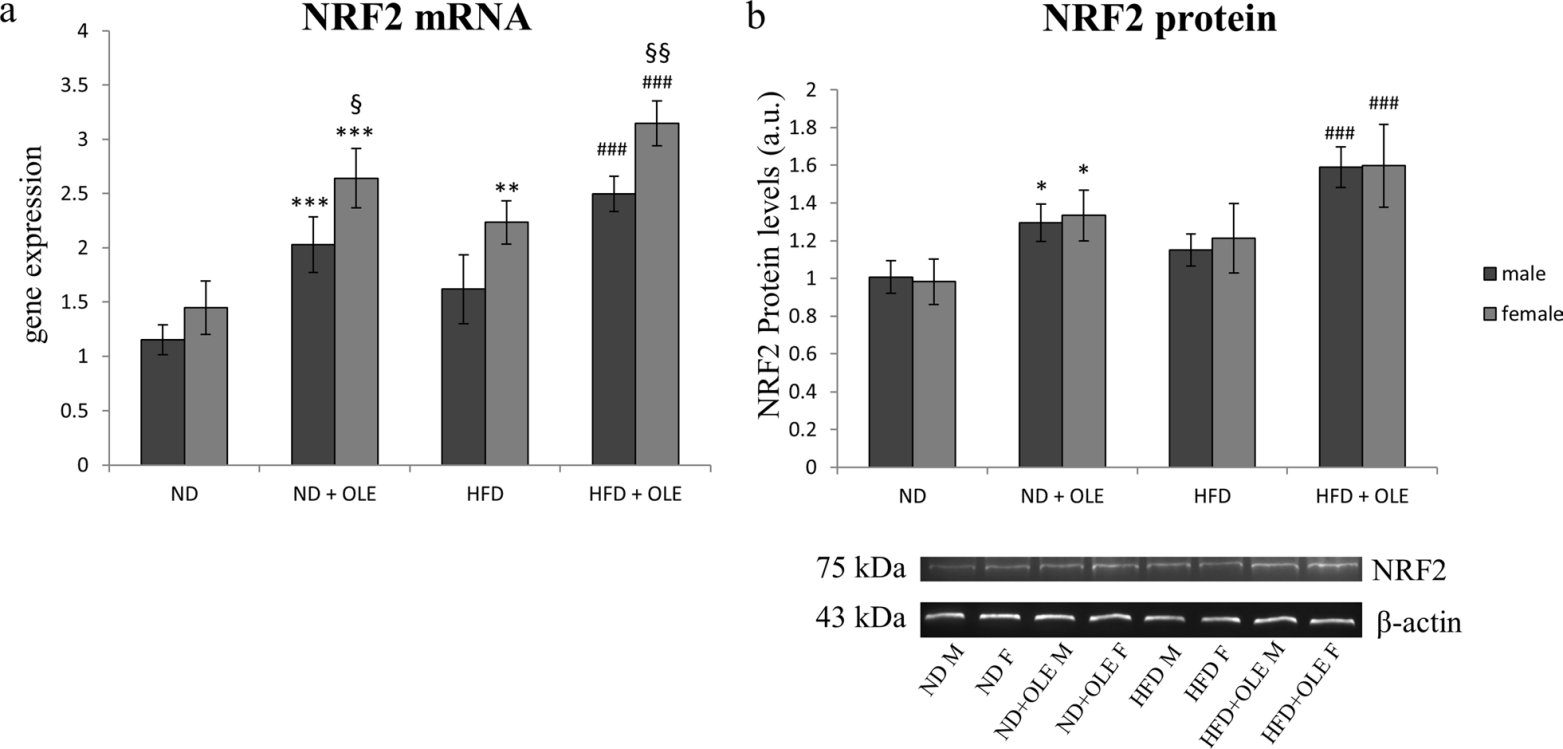
NRF2 levels in ND and HFD mice in presence or absence of Ole. Transcriptional expression (**a**) and protein levels (**b**) of NRF2. Data are expressed as fold mean ± SD and were analyzed by a factorial ANOVA with post-hoc Fisher’s tests for multiple comparison (*P < 0.05, **P < 0.01, ***P < 0.001 vs. ND mice; ^###^*P* < 0.001, vs. HFD mice; ^§^*P* < 0.05; ^§§^*P* < 0.01 vs. male mice)

Nuclear factor E2-related factor 2 (NRF2) is involved in transcriptional modulation in response to copper [49]. Moreover, it is known that the expression of GLO I is regulated by the transcription factor NRF2 [50]. Thus, we analyzed NRF2 transcriptional and translational levels, in the mouse model treated with Ole. Ole administration enhanced both mRNA and protein amount of NRF2 in all the experimental nutritional conditions (Fig. 8). Besides that, sex-related differences were observed: female mice displayed significant higher mRNA levels respect to male (Fig. 8a).

Interestingly, NRF2 transcripts were significantly increased in HFD fed females, highlighting an attempted adaptive response, not followed by an increased in protein levels (Fig. 8b).

## Discussion

Transition metals are known to mediate the autoxidation of glycoprotein, and the oxidation of lipids as well [51]. There are conflicting data on the levels of serum and liver copper observed in NAFLD patients. Although low levels of copper have been described in the serum of NAFLD patients [52–54], copper ions have also been described to increase through cirrhosis to HCC, in MetS and in obese patients, in whose it plays an important role in the accumulation of fat in adipose tissue [28, 55, 56]. The discrepancy between the literature data may be due to several issues, i.e. the heterogeneity of the genetic features of NAFLD patients or the use of different experimental models. By example, Stättermayer et al. have highlighted evident differences in copper liver concentration between NAFLD patients who have the Patatin-like phospholipase domain-containing protein 3 (PNPLA3) mutation associated with presence of NASH and hepatic fibrosis without MetS respect to patients with MetS. The authors claim that MetS might mask the effects of hepatic copper and PNPLA3 [54]. Accordingly, Van Campenhout et al. have shown that even if copper concentration is lower in the early phase of NAFLD disease they were not able to highlight any differences in the progression of the disease [57]. The different findings of copper concentrations might be due to the presence of genetic mutations on the copper transporter genes, such as ATP7B, that could deeply altered copper homeostasis [58]. Further studies are needed to better clarify the copper role in NAFLD taking also into account the different stages of the disease and co-morbidities.

Recent evidence reveals that Cu transport systems have been linked to various pathologies such as hypertension, inflammation, atherosclerosis and diabetes, and play an essential role in the physiological responses of cells, including cell growth, migration, angiogenesis and wound repair [59]. Accordingly, multiple synthetic pathways of carbonyl compounds, from glucose or lipids, require transition metals, such as copper or iron [51]. The oxidative processes, in turn, are involved in the formation of carbonyl compounds, such as glyoxal and MG [60]. Furthermore, copper ions are essential for the activities of semicarbazide sensitive amine oxidase (SSAO), that exerts as a synthetic enzyme for MG [61]. However, it has been also reported that, in adipose tissue, the Cu deficiency is related to a downregulation of SSAO activity, thus more studies are needed to elucidate the relationship between copper and SSAO [62]. Thus, we evaluated the dicarbonyl stress-related liver damage and the concentration of copper in the serum and liver of HFD fed mice. Interestingly, in our mouse model were both altered in presence of liver steatosis, together with the influx and efflux Cu-transporter proteins: CTR1 and ATP7B (Fig. 1).

Interestingly enough, it is well known that the protective and antioxidant effects of the beneficial effects of extra virgin olive oil are affected by the intracellular redox status and oxidative stress [28, 63]. Furthermore, the antioxidant effects of extra virgin olive oil can be attributed to the large presence of polyphenols, and the most abundant among these is Ole, responsible of the organoleptic characteristics of olive oil [64]. It has recently been observed that Ole, in vitro, can form a complex with Cu, acting as a chelating agent [30]. Given that, Ole, as natural chelating agent for copper, could be effective against dicarbonyl stress.

In our hands, Ole, in presence of liver steatosis, was able to further increase the expression levels of CTR1 and ATP7B, let thinking that a higher Cu-dependent antioxidant activity may be induced by the treatment with the nutraceuticals compound (Figs. 3, 4). The positive effects of Ole on the expression levels of Cu-chaperone COX17 (Fig. 5a) could be important for preventing the deregulation of glycolytic flux and the alteration of the mitochondrial metabolism, both involved in the induction of dicarbonyl stress. Accordingly, it has been demonstrated that COX17 controls the Mitochondrial Contact Site and Cristae Organizing System (MICOS), and furthermore COX17-MICOS interaction is regulated by copper ions [65].

On the other hand, it is important to underline that the high levels of CCS transcript and protein expression, induced by Ole treatment, might cause less Cu availability, as the copper could remain seized by CCS chaperone [66]. Moreover, it is interesting to note that it exists important sex-related differences in the protein expression of CCS and ATOX1 in response to Ole treatment, in HFD and ND fed mice. In our knowledge, up to now, any data has been made in the literature in this regard. Further studies are needed to better elucidate the potential differences between male and female in the expression of these two important copper related chaperones after Ole treatment.

After all, the results obtained on CTR2 transcript are quite interesting. In particular, we highlighted that HFD diet has different effects in modulating the two copper transporter proteins: CTR1 and CTR2. The role of CTR2 in Cu homeostasis is not clear, and the effects on CTR2 by Ole treatment are even less known [67]. Further studies are needed to elucidate this point.

Furthermore, Ole, as showed in Fig. 6b, was also able to restores the normal levels of MG in liver tissues of HFD fed mice, and this protective effect well correlates with an increased activity of both GLO I and GLO II (Fig. 7a, b). In addition, the recovery of a redox balance, induced by Ole, has allowed a greater availability of GSH, essential cofactor for the action of GLO I (Figs. 6a, 7a).

In short, Ole has the ability to bind copper and to decrease oxidative stress, these already described mechanisms could justify the better bioavailability of GSH, which is important to carry copper from CTR1 to chaperones [30, 68]. In addition, the increase in GSH levels is critical for the enzymatic activity of GLO I thus it might influences the observed oleuropein dependent decrease of copper-induced toxicity on the metabolic glyoxalases pathways in a virtuous circle way.

Lastly, the master regulator of cellular anti-oxidant, anti-inflammatory and anti-glycation defense systems is NRF2 [69]. It is also known that the development of chronic inflammatory diseases is correlated with a dysregulation of NRF2 activity [70]. Sharma and colleagues recently have indicated NRF2 as a possible treatment for NAFLD/MAFLD and NASH/MASH [71]. NRF2 is involved in the deregulation of many pathways, including fatty acid oxidation, glucose metabolism, antioxidant response and dicarbonyl stress response [72]. Moreover, as reported above, we have already shown that Ole is able to induce AMPK-dependent autophagy process. Accordingly, Mo et al. [73] demonstrated that the activation of AMPK together with the NRF2 pathways improve anti-inflammatory effects, oxidative stress/proinflammatory response and lipid metabolism in NAFLD, confirming the reliability of our results. Finally, NAFLD/MAFLD progression toward NASH/MASH was slowed down in mice treated with NRF2 activators [69].

Interestingly, our data suggest that Ole might carry out its beneficial effect both by preventing the accumulation of copper and by decreasing dicarbonyl stress through the activation of NRF2 dependent pathways (Fig. 8).

The different sensitivity to Ole, observed between male and female mice, in the expression of Cu-transporter proteins and in the modulation of the defense system of glyoxalases, may have a hormonal basis [74]. Accordingly, in vitro studies have shown that down regulation of ATP7B expression increased copper levels less markedly in the presence of estrogen [75, 76]. Furthermore, Rulli et al. highlighted a modulatory role of estradiol in the expression of both glyoxalases I and II, in in vitro model [77]. Again, until the period of menopause the prevalence and severity of NAFLD/MAFLD is lower in women than in men.

## Conclusions

**Fig. 9:**
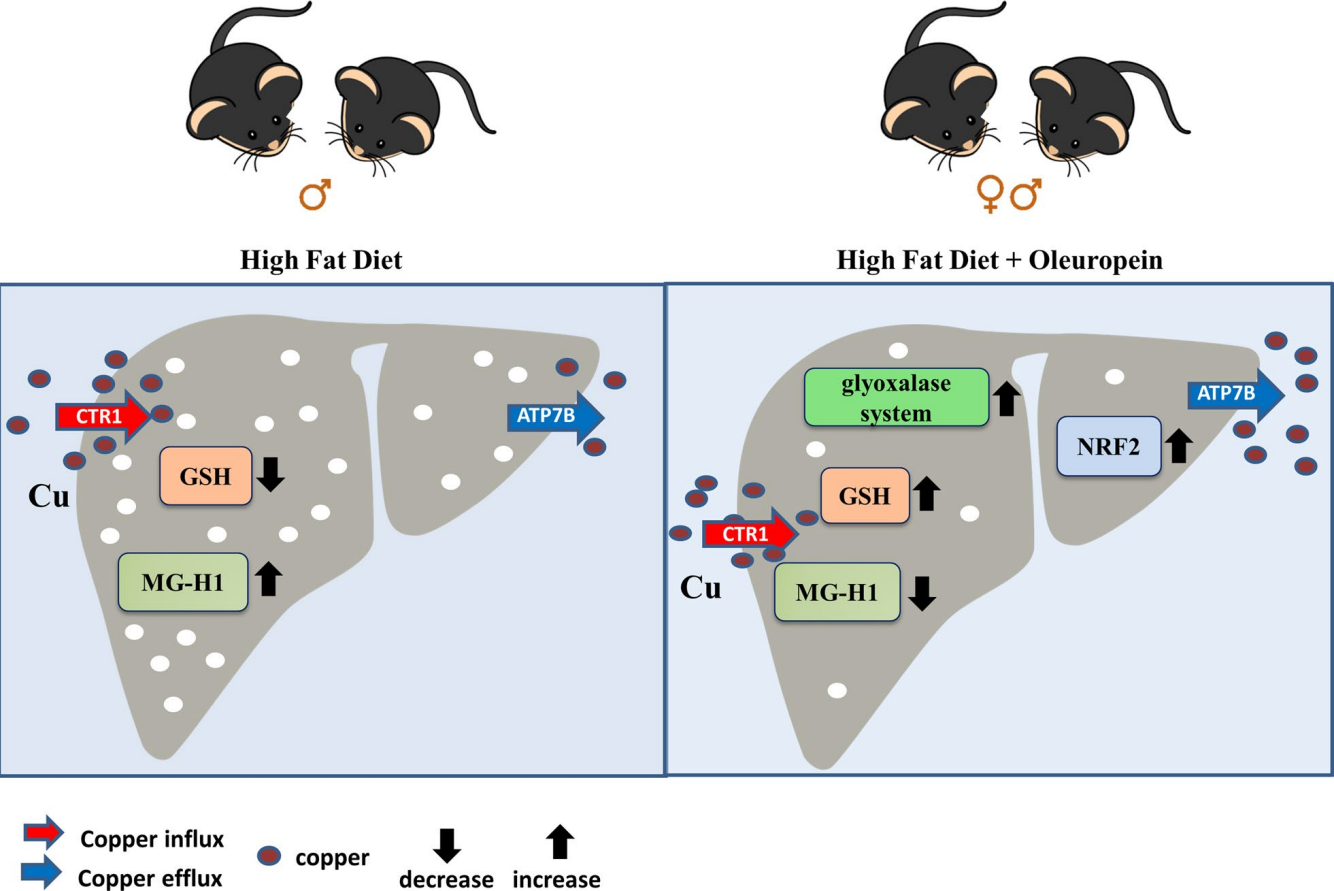
Graphical abstract. Graphical representation of HFD and Ole effects in mice fed ND or HFD. The red and blue arrows indicate CTR1 and ATP7B copper transporter proteins, respectively

Our work demonstrates that copper homeostasis has a key role in the HFD-related methylglyoxal pathway derangement and the early administration of Ole, acting as a natural copper chelator, could be able to protect liver from fat pro-oxidant and pro-glycative effects (Fig. 9). Clinical trial designed to clarify if early intake of Ole could impair the progression of NAFLD/MAFLD toward NASH/MASH are needed.

## Supplementary Information

**Additional file 1.** **Table S1.** Weight and biochemical parameters of ND and HFD mice treated or not with Ole. The results are represented as mean values ± SD. (* P<0.05, ** P< 0.01, *** P < 0.001 vs ND mice; # P < 0.05, ## P < 0.01; ### P < 0.001 vs HFD mice; § P < 0.05, §§ P < 0.01, §§§ P < 0.001 vs male mice).

**Additional file 2.** **Fig. S1_supplementary.** Oleuropein decreases copper levels in hepatoma cell lines. Copper concentration in HepG2 cells treated for 24 hrs with FAs (0.5 mM) and increasing dosages of Ole (0–200 µM). The results, derived from four independent experiments, and are represented as mean ± SD. (** P < 0.01; *** P < 0.001 vs control).

## Data Availability

The data that support the findings of this study are available from the corresponding author, upon reasonable request.

## Abbreviations

NAFLD: Nonalcoholic fatty liver disease
MetS: Metabolic syndrome
MAFLD: Metabolic associated fatty liver disease
NASH: Nonalcoholic steatohepatitis
MASH: Metabolic-associated steatohepatitis
MG: Methylglyoxal
Ole: Oleuropein
HFD: High-fat diet
ND: Normal diet
MG-H1: Methyl-glyoxal-hydro-imidazolone
