# Supplementary material for: Copper-catalyzed dicarbonyl stress in NAFLD mice: protective effects of Oleuropein treatment on liver damage
Source: Nutr Metab (Lond). 2022 Feb 11;19:9. doi: 10.1186/s12986-022-00641-z (PMC8832663; doi:10.1186/s12986-022-00641-z)
Supplement: Supplementary file 1 — Additional file 1. Table S1. Weight and biochemical parameters of ND and HFD mice treated or not with Ole. The results are represented as mean values ± SD. (* P<0.05, ** P< 0.01, *** P < 0.001 vs ND mice; # P < 0.05, ## P < 0.01; ### P < 0.001 vs HFD mice; § P < 0.05, §§ P < 0.01, §§§ P < 0.001 vs male mice). [file 12986_2022_641_MOESM1_ESM.docx]

| ***Sex*** | **Group** | **Body weight at the start of treatment**  **(g)** | **Body weight at the end of treatment**  **(g)** | **Liver weight**  **(g)** | **ALT (U/L)** | **AST (U/L)** | **Total cholesterol (mg/dL)** | **HDL-c (mg/dL)** | **LDL-c (mg/dL)** | **TRIG (mg/dL)** |
| --- | --- | --- | --- | --- | --- | --- | --- | --- | --- | --- |
| ***Males***  ***(n 16)*** | **ND**  **(n 4)** | 25.4 ± 1.6 | 32.8 ± 2.1 | 1.7 ± 0.2 | 49.8 ± 1.5 | 105.4 ± 25.1 | 163.4 ± 10.5 | 84.2 ± 2.2 | 12.3 ± 3.6 | 125.3 ± 5.5 |
|  | **ND+OLE**  **(n 4)** | 25.3 ± 1.6 | 32.9 ± 3.2 | 1.6 ± 0.8 | 48.8± 1.9 | 102.6 ± 21.3 | 152.1 ± 5.7 | 88.6 ± 2.4 | 10.5 ± 0.8 | 119.1 ± 3.2 |
|  | **HFD**  **(n 4)** | 25.1 ± 1.8 | **47.6 ± 3.1^**^** | **4.5 ± 0.3^**^** | **122.9 ± 23.1^**^** | **206.4 ± 19.6^*^** | **203.2 ± 12.8^*^** | **125.5 ± 2.5^***^** | 14.4 ± 3.2 | 133.2 ± 3.7 |
|  | **HFD+OLE**  **(n 4)** | 25.2 ± 1.7 | 38.7 ± 3.1 | 2.8 ± 0.4 | **84.2 ± 5.3^##^** | **111.4 ± 9.6^##^** | **168.9 ± 9.8^#^** | **89 ± 1.8^###^** | 12.1 ± 2.7 | 122.5 ± 4.5 |
| ***Females***  ***(n 16)*** | **ND**  **(n 4)** | 19.7 ± 1.3 | 23.9 ± 1.3 | 1.0 ± 0.4 | 45.6 ± 2.5 | 102.1 ± 12.5 | 154,1 ± 9.4 | 81.6 ± 2.8 | 10.4 ± 1.3 | **112.5 ± 4.3^§§§^** |
|  | **ND+OLE**  **(n 4)** | 19.9 ± 1.1 | 24.7 ± 1.9 | 1.2±0.3 | 42.2 ± 16.4 | 89.8 ± 6.5 | 150.1 ± 3.4 | 88.1 ± 2.3 | 11.9 ± 1.3 | **98.6 ± 4.1^§§^** |
|  | **HFD**  **(n 4)** | 19.6 ± 1.7 | 34.6 ± 2.3 | 2.8 ± 0.8 | **79.5 ± 3.2^***,§^** | **198.2 ± 4.2^**^** | **200.1 ± 1.6^**^** | **116.5 ± 1.4^***^** | 11.8 ± 0.3 | **108.2 ± 4.6§** |
|  | **HFD+OLE**  **(n 4)** | 19.6 ± 1.5 | 32.2 ± 1.4 | 1.9 ± 0.8 | **52.1 ± 3.2^###,§§^** | **105.4 ± 10.4^###^** | **167.1 ± 2.2^###^** | **85.2 ± 2.2^###^** | 9.1 ± 0.9 | **99.6 ± 1.6^§§^** |

**Table S1**: Weight and biochemical parameters of ND and HFD mice treated or not with Ole. The results are represented as mean values ± SD. (* P < 0.05, ** P < 0.01, *** P < 0.001 vs ND mice; # P < 0.05, ## P < 0.01; ### P < 0.001 vs HFD mice; § P < 0.05, §§ P < 0.01, §§§ P < 0.001 vs male mice).
