# Supplementary figures and images for: Copper-catalyzed dicarbonyl stress in NAFLD mice: protective effects of Oleuropein treatment on liver damage
Source: Nutr Metab (Lond). 2022 Feb 11;19:9. doi: 10.1186/s12986-022-00641-z (PMC8832663; doi:10.1186/s12986-022-00641-z)

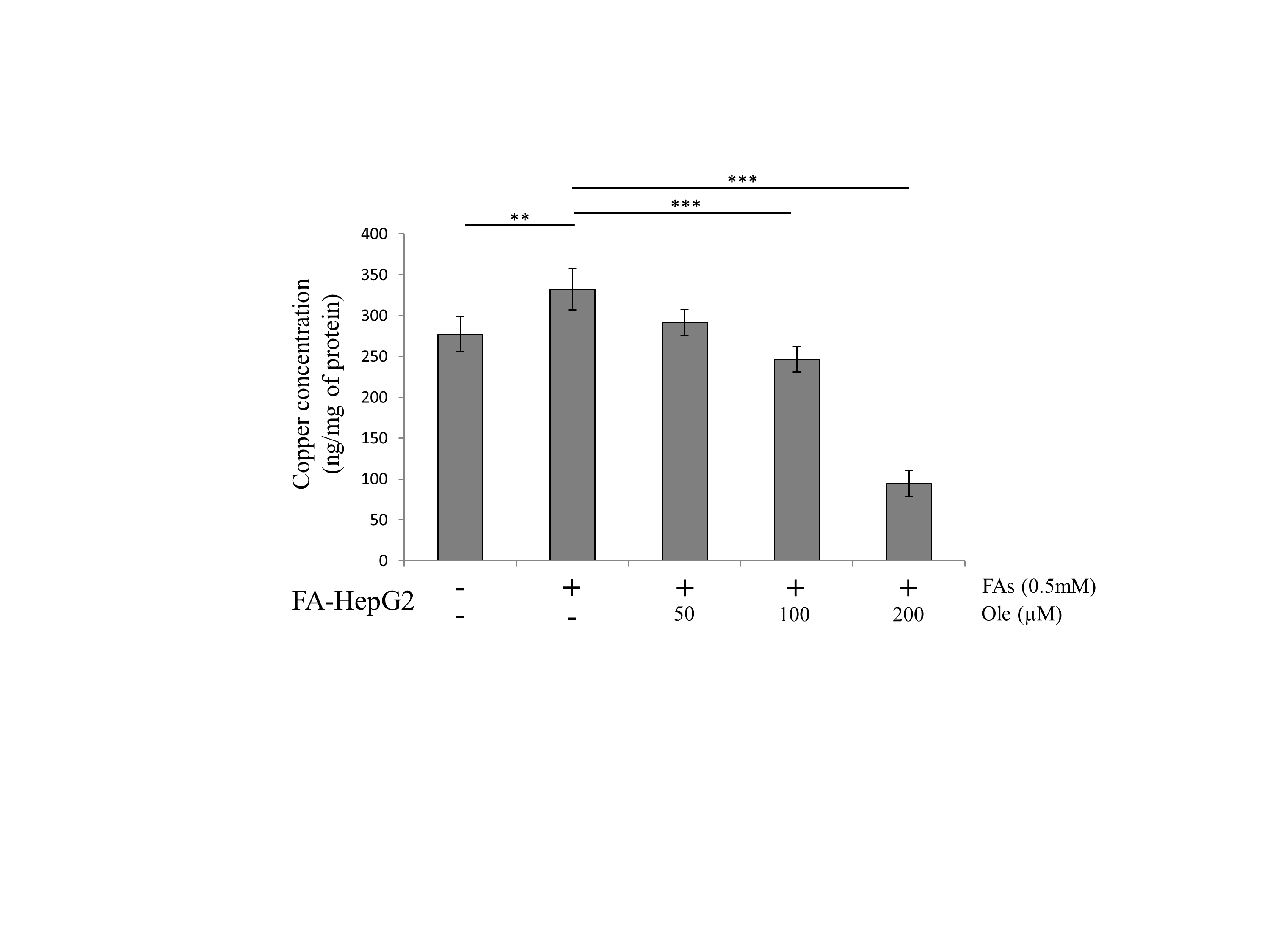

Supplement: Supplementary file 2 — Additional file 2. Fig. S1_supplementary. Oleuropein decreases copper levels in hepatoma cell lines. Copper concentration in HepG2 cells treated for 24 hrs with FAs (0.5 mM) and increasing dosages of Ole (0–200 µM). The results, derived from four independent experiments, and are represented as mean ± SD. (** P < 0.01; *** P < 0.001 vs control). [file 12986_2022_641_MOESM2_ESM.tif]
